# Autonomic control of the heart: going beyond the classical neurotransmitters

**DOI:** 10.1113/expphysiol.2014.080184

**Published:** 2014-11-20

**Authors:** Neil Herring

**Affiliations:** Burdon Sanderson Cardiac Science Centre, Department of Physiology, Anatomy and Genetics, University of OxfordOxford, OX1 3PT, UK

## Abstract

Acute myocardial infarction and congestive cardiac failure are characterized by high levels of cardiac sympathetic drive. In these conditions, sympathetic neurotransmitters such as neuropeptide Y (NPY) can be released in addition to noradrenaline, and plasma levels correlate with infarct size and mortality. Even in the presence of β-blockers, NPY is able to bind to its own receptors located on cholinergic ganglia and ventricular myocytes. In this symposium report, I review the evidence that NPY can inhibit acetylcholine release during vagus nerve stimulation and limit the subsequent bradycardia. I also present preliminary, as yet unpublished data, demonstrating that NPY may be pro-arrhythmic by directly influencing ventricular electrophysiology. Targeting NPY receptors pharmacologically may therefore be a useful therapeutic strategy both to reduce heart rate and to prevent arrhythmias in the setting of myocardial infarction and chronic heart failure. Such medications would be expected to act synergistically with β-blockers, angiotensin-converting enzyme inhibitors and implantable cardiac devices, such as defibrillators and vagus nerve stimulators.

**New Findings**

- What is the topic of this review?
  This symposium report discusses the evidence for release of cardiac sympathetic cotransmitters in addition to noradrenaline.
- What advances does it highlight?
  It highlights the potential role of neuropeptide Y in reducing vagal neurotransmission and directly influencing ventricular myocyte excitability in the presence of β-receptor blockade.

## Introduction

β-Blockers, introduced over 50 years ago, are the only anti-arrhythmic drug proven to improve mortality after myocardial infarction and in chronic congestive heart failure, where sympathetic drive to the heart is high (ISIS-1, 1986; CIBIS-II, 1999). We have previously demonstrated that high-level sympathetic stimulation causes the release of sympathetic neurotransmitters in addition to noradrenaline (NA), such as neuropeptide Y (NPY). In this symposium report, I present preliminary data suggesting that even in the presence of β-blockers, the release of additional sympathetic cotransmitters can have deleterious consequences for cardiac function.

## Neuropeptide Y and sympathovagal cross-talk

A long-lasting inhibition of cardiac vagal function following high levels of cardiac sympathetic stimulation in the presence of β-blockade was first observed *in vivo* as early as 1982 (Potter, 1982). Our group has explored this phenomenon *in vitro* using an isolated atrial preparation with intact right stellate ganglion and right vagus nerve to enable neuronal stimulation and pharmacological manipulation to be performed independent of changes in haemodynamics and other circulating factors. Interestingly, impaired cholinergic regulation of heart rate following adrenergic stimulation *in vitro* requires direct stimulation of the sympathetic and vagus nerves and is not observed with exogenous NA and/or acetylcholine (Herring *et al*. 2012). This suggests that the phenomenon is dependent on interneuronal signalling via an additional neurotransmitter.

Geoffrey Burnstock was the first to suggest that neurons release more than one functional neurotransmitter (Burnstock, 1976). Henry Dale's principle was subsequently redefined by Sir John Eccles to state that ‘at all the axonal branches of a neuron, there is liberation of the same transmitter substance *or substances*’ to incorporate Burnstock's theory that nerves of the same class use more than one neurotransmitter (Eccles, 1976). Significant cotransmitter release generally occurs only on high-level neuronal stimulation, where they act as slowly diffusing molecules. Sympathetic nerves throughout the autonomic nervous system contain cotransmitters such as ATP and NPY in addition to NA. Whilst NPY has a long biological half-life, ATP is rapidly metabolized (Burnstock, 2009). Moreover, neither NA nor ATP influences cardiac vagal acetylcholine release within human or guinea-pig heart (Manabe *et al*. 1991; Schwertfeger *et al*. 2004). Levels of sympathetic stimulation identical to those that impair vagal function have been shown to release NPY into the perfusate of isolated atria *in vitro* (Herring *et al*. 2012) as well as into coronary sinus blood *in vivo* (Warner *et al*. 1991). Antagonists of Y_2_ receptors (Smith-White *et al*. 2001; Herring *et al*. 2012) or genetic knockout of these receptors (Smith-White *et al*. 2002) can prevent the inhibition of vagal bradycardia after sympathetic stimulation. All of these experiments are performed in the presence of a β-blocker, and it is possible that β-blockers may inhibit the release of NA and NPY. Whilst presynaptic α_2_ receptors are involved in autoinhibition of NA release, neuronal β_1_ and β_2_ receptors may facilitate release. The clinical use of β-blockers after myocardial infarction and during chronic heart failure is unlikely to change given their mortality benefit, and it is therefore wise to study potential new therapies in combination with their use. It is also possible that NPY itself may autoinhibit or stimulate its own release and that of NA, although this also is yet to be demonstrated in the heart.

As well as being present in sympathetic neurons originating from the stellate ganglia, NPY is found in a variety of intrinsic cardiac ganglia cells, which may be sensory, sympathetic or even interneurons. Immunohistochemical labelling has also localized the Y_2_ receptor to cholinergic vagal neurons in the right atrium and around the sinoatrial node (Herring *et al*. 2008), providing the anatomical basis for the sympathovagal cross-talk model. It may be that cross-talk is more complex than NPY simply acting on postganglionic cholinergic end terminals given the potential for other sympathetic and sensory interactions with parasympathetic transmission within the network of intrinsic cardiac ganglia.

Exogenous NPY, whilst having no direct effect on heart rate itself, can reduce the heart rate response to vagus nerve stimulation *in vitro*. Over the same concentration range, it is unable to alter the bradycardic response to the stable acetylcholine analogue, carbamylcholine, but significantly reduces [^3^H]-acetycholine release during neuronal stimulation (Herring *et al*. 2008). Intravenous administration of NPY is also able reduce the magnitude of the vagal bradycardia *in vivo* (Potter, 1987; Warner & Levy, 1989). In our atrial preparation with intact right vagus nerve, inhibitors of protein kinase C completely abolish the action of NPY, whilst protein kinase A inhibitors have no effects. Conversely, a protein kinase C activator can mimic the effects of NPY and reduce [^3^H]-acetycholine release (Herring *et al*. 2008). The exact mechanisms linking Y_2_ receptor binding, protein kinase C signalling and the release of acetylcholine have not yet been elucidated. In the spontaneously hypertensive rat, an animal model of genetic hypertension, even before the development of the hypertensive phenotype, high levels of cardiac sympathetic drive and NPY release can be observed, leading to a resting tachycardia (Shanks *et al*. 2013).

By lowering the heart rate, the vagus reduces cardiac metabolic demand and prevents intracellular calcium overload. Independent of heart rate, the vagus can also raise the threshold for induction of ventricular arrhythmias (Nash *et al*. 2001), potentially through a neuronal nitric oxide-dependent pathway (Brack *et al*. 2007). Unsurprisingly, impaired vagal function is a negative prognostic indicator in conditions such as congestive cardiac failure (La Rovere *et al*. 1998; Cole *et al*. 1999). Although NPY influences vagal control of heart rate, which in itself could be therapeutically beneficial, our preliminary data suggest that independent of these heart rate changes, cholinergic modulation of ventricular fibrillation (VF) threshold (VFT) remains intact in the presence of NPY.

## A direct action of NPY on cardiac excitability?

The role of NPY in arrhythmogenesis may extend beyond sympathovagal cross-talk, because NPY receptors, particularly Y_1_ receptors, are also expressed on ventricular myocytes in both rats (Chottová Dvoráková *et al*. 2008) and humans (Jönsson-Rylander *et al*. 2003). The action of NPY on ventricular myocyte electrophysiology is complex, with a variety of results being reported in single-cell experiments depending on whether NPY is administered alone or in combination with β-adrenergic stimulation. Neuropeptide Y does not appear to alter the voltage-gated sodium current (*I*_Na_) or the delayed rectifier potassium current (*I*_K_; Zhao *et al*. 2006) but can reduce the transient outward potassium current (*I*_to_) via an inhibitory G-protein-sensitive pathway (Heredia *et al*. 2002). Neuropeptide Y appears to inhibit the L-type calcium current (*I*_CaL_) in the guinea-pig (Bryant & Hart, 1996), which may explain its negative contractile action in this species (which lacks *I*_to_), but stimulates this current in the rat (Millar *et al*. 1991). In addition, NPY can increase cell shortening and the size of the calcium transient in rat ventricular myocytes via a Y_1_ receptor pathway coupled to activation of phospholipase C (Heredia Mdel *et al*. 2005). Can NPY released during high-level sympathetic nerve stimulation in the presence of a β-blocker therefore be arryhthmogenic by acting directly on the myocardium, leading to intracellular calcium loading?

As part of the symposium presentation, I presented unpublished data suggesting that NPY may play an important role by acting directly on ventricular myocytes to increase susceptibility to ventricular fibrillation. Dr Manish Kalla and I have developed a Langendorff-perfused rat heart with intact innervation, enabling simultaneous or individual stimulation of each stellate ganglion. The susceptibility of the heart to VF can be evaluated after high-frequency burst pacing at increasing current amplitude following a pacing drive train so that VFT can be measured accurately independent of heart rate. Ventricular fibrillation is cardioverted with a bolus of high-concentration potassium chloride solution, and control experiments demonstrate that VFT remains constant over consecutive inductions. Whilst a maximal concentration of NA lowers the VFT, metoprolol can completely block its effect, demonstrating its efficacy as an antiarrhythmic β-blocker. However, metoprolol is unable to prevent the fall in VFT following prolonged high-frequency stimulation of either the right or the left stellate ganglion, despite the chronotropic effect being completely abolished. This suggests that an additional substance released from sympathetic nerve endings during these conditions may also be acting as a pro-arrhythmic signal. Consistent with this hypothesis, exogenous NPY also directly lowers VFT by a Y_1_-dependent mechanism. Combining a Y_1_ receptor antagonist with metoprolol also prevents the fall in VFT following high-frequency stellate stimulation. Optical mapping experiments in the isolated rat heart using a voltage-sensitive dye also show that NPY steepens the action potential duration restitution curve, demonstrating a direct pro-arrhythmic action on ventricular electrophysiology. Combining β-blockade with an antagonist of the NPY Y_1_ receptor may therefore be a useful strategy for preventing arrhythmias in conditions where cardiac sympathetic drive and NPY release are high.

Several studies published over 20 years ago demonstrate that plasma NPY levels are elevated following acute coronary syndromes and during left ventricular failure in humans where they correlate positively with the severity of heart failure and 1 year mortality (Hulting *et al*. 1990; Ullman *et al*. 1994). We have also recently characterized plasma levels of NPY following ST-elevation myocardial infarction treated with primary percutaneous coronary intervention. At presentation, plasma levels were significantly elevated, and despite emergency contemporary treatment, remained elevated even after 48 h of treatment (Cuculi *et al*. 2013). Given the persistent sympathetic hyperactivity observed during chronic heart failure and the long plasma half-life of NPY, its neuromodulator actions described in this review may well be a persistent feature of this condition.

## Concluding comments

**Figure 1:**
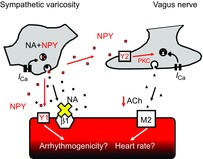
Potential actions of the sympathetic cotransmitter neuropeptide Y (NPY, in red) Although β-blockers (represented by the yellow cross) prevent the action of noradrenaline (NA, in black), high-level sympathetic stimulation also causes the release NPY. Neuropeptide Y can act on Y_2_ receptors on cholinergic ganglia to reduce the release of acetylcholine (ACh) and result in vagal bradyacardia via a protein kinase C (PKC)-dependent pathway. Independent of heart rate, NPY may also act on Y_1_ receptors on ventricular myocytes to influence their electrophysiology and predispose to ventricular arrhythmias. Adapted with permission from Herring *et al*. (2012).

Targeting NPY receptors pharmacologically may therefore be a useful therapeutic strategy after myocardial infarction. Targeting the Y_2_ receptor may facilitate vagal bradycardia and reduce resting heart rate, whilst Y_1_ receptor inhibition may prevent ventricular arrhythmias independent of heart rate (see Fig.1). Such medications would be expected to act synergistically with β-blockers, angiotensin-converting enzyme inhibitors and implantable cardiac devices, such as defibrillators and vagus nerve stimulators.

## Call for comments

Readers are invited to give their opinion on this article. To submit a comment, go to: http://ep.physoc.org/letters/submit/expphysiol;100/4/354
